# LncRNA Snhg1 Plays an Important Role *via* Sequestering rno-miR-139-5p to Function as a ceRNA in Acute Rejection After Rat Liver Transplantation Based on the Bioinformatics Analysis

**DOI:** 10.3389/fgene.2022.827193

**Published:** 2022-06-02

**Authors:** Wu Wu, Menghao Wang, Chunming Li, Zhu Zhu, Yang Zhang, Di Wu, Zhibing Ou, Zuojin Liu

**Affiliations:** ^1^ Department of Hepatobiliary Surgery, The Second Affiliated Hospital of Chongqing Medical University, Chongqing, China; ^2^ Department of Gastrointestinal Surgery, Chengdu Seventh People’s Hospital, Chengdu, China; ^3^ Key Laboratory of Hepatobiliary and Pancreatic Surgery, Institute of Hepatobiliary Surgery, Southwest Hospital, Third Military Medical University, Chongqing, China; ^4^ Department of Hepatobiliary Surgery, Chenzhou No.1 People’s Hospital, Chenzhou, China

**Keywords:** ceRNA network, liver transplantation, acute rejection, SNHG1, rno-miR-139-5p

## Abstract

In order to explore the molecular mechanism of acute rejection after liver transplantation (ARLT) in rats, we employed the GSE36798 data set in the Gene Expression Omnibust (GEO) database to construct a related ceRNA network. This dataset contained a total of 16 samples (8 graft samples and 8 plasma samples). Each kind of sample was divided into acute rejection (AR) groups and non-acute rejection (NR) groups, and each group had 4 replicates. First, we performed principal component analysis (PCA) with downloaded data to compare the difference between samples in a macroscopic way. Then, we used the “limma” R package to screen out differentially expressed miRNAs among different groups and used the “pheatmap” R package to perform bidirectional hierarchical clustering analysis for these differentially expressed miRNAs. The miRWalk database and the LncBase V.2 database were applied to predict downstream target genes and upstream-related lncRNAs, respectively. Meanwhile, the String database was used to predict the relationship between target genes, and the aforementioned results were processed for visualization by Cytoscape software. In addition, we exhibited the ultimate ceRNA network, including two lncRNAs, two miRNAs, and 77 mRNAs. Finally, we constructed a rat model of ARLT and applied graft specimens to relevant experimental verification. We found that the lncRNA Snhg1/rno-miR-139-5p axis might be involved in the regulation of ARLT in rats. In short, we demonstrated the differentially expressed miRNA profile, constructed a related ceRNA network, and screened out a possible regulatory axis. In view of the conservation of genes among species, this work was expected to provide a new strategy for the treatment and prevention of ARLT in the clinical setting.

## Background

In the past few decades, liver transplantation has been the fastest growing approach in the field of organ transplantation. When various acute or chronic liver diseases cannot be cured by surgery and other medical methods, so that death cannot be avoided in a short term, liver transplantation will become the only effective treatment method (Jadlowiec and Taner, 2016; Mazzaferro et al., 2020). It is a miracle created by modern medicine to save patients with end-stage liver disease by transplanting all or part of the liver. However, complications regarding ARLT and loss of graft function are currently important obstacles affecting the long-term survival of patients. Acute rejection is the most common complication in the early stages after liver transplantation. The HLA antigen of the transplant stimulates the recipient’s T lymphocytes to differentiate and proliferate, producing a large number of specific sensitized lymphocytes, which can directly kill or release various lymphokines to kill target cells (Davis and Cooper, 2017; Lee, 2017). With the emergence of various new immunosuppressive drugs, the incidence of severe acute rejection in clinical trials has been greatly reduced, and the survival rate of patients has been significantly improved. However, our understanding of the underlying molecular mechanism of ARLT is still insufficient. Further exploration is rather necessary for improving the success rate of liver transplantation and for extending the survival time of patients after transplantation.

Non-coding RNA (ncRNA) refers to RNA that does not encode protein, including rRNA, lncRNA, and microRNA (Dragomir et al., 2020; Panni et al., 2020). Even if these RNAs are not translated into proteins, they can drive specific cellular biological responses through molecular targets (Qian et al., 2019). Furthermore, they play key regulatory roles in many biological processes of organisms, including epigenetics, transcription, post-transcriptional modification, and signal transduction, which exhibit important physiological significance. Competitive endogenous RNA (ceRNA) has attracted much attention from academia in recent years, and it represents a brand new mode of gene expression regulation (Smillie et al., 2018). Compared with the simple miRNA regulatory network, the ceRNA regulatory network is more sophisticated and complex, in which more RNA molecules are involved, including mRNA, pseudogenes of coding genes, lncRNAs, and miRNAs. The theoretical core of ceRNA refers to the fact that there exist competitive endogenous RNAs in cells, and these ceRNA molecules can compete with the same miRNA through the miRNA response element (MRE) to adjust expression levels (Thomson and Dinger, 2016; Zhong et al., 2018).

Recently, some research studies regarding the ceRNA network have shown that it participates in the regulation of many physiological processes and relevant diseases, such as tumors (Wang et al., 2017; Yang et al., 2018; Tang et al., 2019; Zhang and Zhou, 2020), acute kidney injury (Cheng and Wang, 2020), retinopathy (Yao et al., 2020), limb ischemia (He et al., 2019), and Alzheimer’s disease (Ma et al., 2020; Zhang and Wang, 2021). However, there are few studies for ceRNA to modulate ARLT, and the specific molecular mechanism is still very vague. Therefore, to further deepen our understanding of ARLT regulated by ceRNA, we downloaded the GSE36798 dataset from the GEO database. The author performed the AR model of orthotopic liver transplantation in rats. Brown Norway (BN) rats (recipients) and Lewis rats (donors) were used as the experimental group (Lewis to BN) for orthotopic liver transplantation (OLT); then, BN rats (recipients) and BN rats (donors) were used as the control group (BN to BN). The overall microRNA expression profile of plasma and graft samples was evaluated by microarray. We further analyzed the dataset to construct the related ceRNA network, which provided a theoretical basis for elucidating the molecular mechanism of ARLT and for finding new therapeutic targets.

## Materials and Methods

### Microarray Data Acquisition From GEO

The GSE36798 dataset (public on 26 Dec 2020) was downloaded from GEO (https://www.ncbi.nlm.nih.gov/gds/?term=). The dataset included a total of 16 samples, including eight tissue samples and eight plasma samples. In each kind of sample, four cases with AR after transplantation were referred to as the experimental group, and four cases with NR were used as the control group. All samples were sequenced using the GPL10906 platform (Agilent-019159 Rat miRNA Microarray, miRBase release 12.0 miRNA ID version).

### Principal Component Analysis (PCA)

“GEOquery” (R package) was used to download the dataset and extract the corresponding expression matrix and clinical information. The soft file of the platform GPL10906 was extracted, and the probe information was mapped into gene information for ID conversion. Finally, two R packages “FactoMineR” and “factoextra” were applied to analyze, describe, and visualize the differences between groups.

### Screening for Differentially Expressed miRNAs and Bidirectional Hierarchical Cluster Analysis

Under the strict criterion (FC >1, *p* <0.05), we used the “limma” R package (Version 3.26.9) to distinguish differentially expressed miRNAs, which were considered as DEGs for subsequent analysis. The differentially expressed miRNAs we screened out were visualized *via* a volcano map, which was executed with two R packages of “dplyr” and “ggplot2”. According to different grouping information, we applied differentially expressed miRNAs between groups for bidirectional hierarchical clustering analysis with the R package “pheatmap”.

### GO and KEGG Functional Enrichment Analysis

All the target genes of differentially expressed miRNAs were imported into the “DAVID” Bioinformatics Resources 6.8 website (https://david.ncifcrf.gov/home.jsp), and the corresponding files were downloaded for visual analysis using the R package “ggplot2”. Under the criterion (*p* <0.05), Gene Ontology (GO) analysis was performed according to the classification of molecular function (MF), biological process (BP), and cell components (CC), and Kyoto Encyclopedia of Genes Genomes (KEGG) analysis was performed according to different signal pathways.

### Construction of Networks

We used the miRWalk online database (http://mirwalk.umm.uni-heidelberg.de/) to predict the whole target genes of differentially expressed miRNAs. In addition, we combined miRDB (http://mirdb.org/), miRBase (http://www.mirbase.org/), TargetScan (http://www.targetscan.org/vert_72/), and other databases to assist in analysis and prediction. The R package “dplyr” was used to prepare the network construction files, and then, Cytoscape software was applied to visualize the miRNA-mRNA network. We predicted the interaction between all target genes to construct a protein–protein interaction (PPI) network through the String online website (https://string-db.org/) with high confidence (0.900). We applied the LncBase V.2 database (https://diana.e-ce.uth.gr/lncbasev3) to predict the relationship between miRNAs and LncRNAs and combined it with the miRNA-mRNA network to construct the lncRNA–miRNA–mRNA network. Then, we used RStudio to prepare input files which were imported into Cytoscape software to drive the visualization.

### Animal and Liver Transplantation Models

Lewis and BN rats (male, 250–300 g) were purchased from the Chongqing Medical University Experimental Animal Centre. The whole rats were raised in an SPF level chamber with a 12-h light/dark cycle at 24°C and 60% humidity and were freely provided with food and water. Animal experiments have been approved by the Biomedical Ethics Committee of Chongqing Medical University. The establishment of animal models is based on the modified Kamada’s two-sleeve technique (Kamada and Calne, 1983). All the samples were obtained on the 7th day after liver transplantation. The detailed information about the model establishment was rooted in the study published by our team (Cheng et al., 2019).

### HE Staining and Quantitative RT-PCR

Graft samples (6um) were stained with hematoxylin and eosin (HE). TRIzol (Sigma) reagent was used to extract total RNA from graft samples. For the analysis, 2 μg total RNA was reversely transcribed into cDNA with the miScript II RT kit (TaKaRa Biotechnology Co., Ltd., Dalian, China), and qPCR was performed with TB Green® Premix Ex Taq™(RR820A, Takara, Japan) on the BioRad real-time PCR instrument. The primer sequences are shown in the Table1.

**TABLE 1:** The primer sequences for qPCR.

Primer name	Primer direction	Primer sequence (5’–3’)
GAPDH	Forward	TCA​ACG​GGG​GAC​ATA​AAA​GT
—	Reverse	TGC​ATT​GTT​TTA​CCA​GTG​TCA​A
TNF-α	Forward	CGC​CAC​GAG​CAG​GAA​TGA​GAA​G
—	Reverse	GGA​AGC​GTA​CCT​ACA​GAC​TAT​C
IL-1β	Forward	AAA​TGA​ACC​GAG​AAG​TGG​TGT​T
—	Reverse	TTC​CAT​ATT​CCT​CTT​GGG​GTA​GA
IL-6	Forward	GTT​CTC​TGG​GAA​ATC​GTG​GA
—	Reverse	TGTACTCCAGGTAGCTA
rno-miR-342-3p	Forward	TGCCCACGCTAAAGACA
—	Reverse	TGGTGTCGTGGAGTCG
rno-miR-139-5p	Forward	GACCTCTGTGCACGTG
—	Reverse	TGGTGTCGTGGAGTCG
U6	Forward	AAC​GCT​TCA​CGA​ATT​TGC​GT
—	Reverse	CTCGCTTCGGCAGCACA
lncRNA Snhg1	Forward	ACA​AGA​GCT​TAC​TGG​TGA​AG
—	Reverse	CAG​GGT​GAA​TAC​AGG​TAT​TC
lncRNA Gm12511	Forward	TCC​ACC​TTC​TGA​GAG​CTA​GG
—	Reverse	ATT​TCC​CCA​CTG​AGA​TCT​TG

## Results

### Principal Component Analysis of Different Groups

**FIGURE 1:**
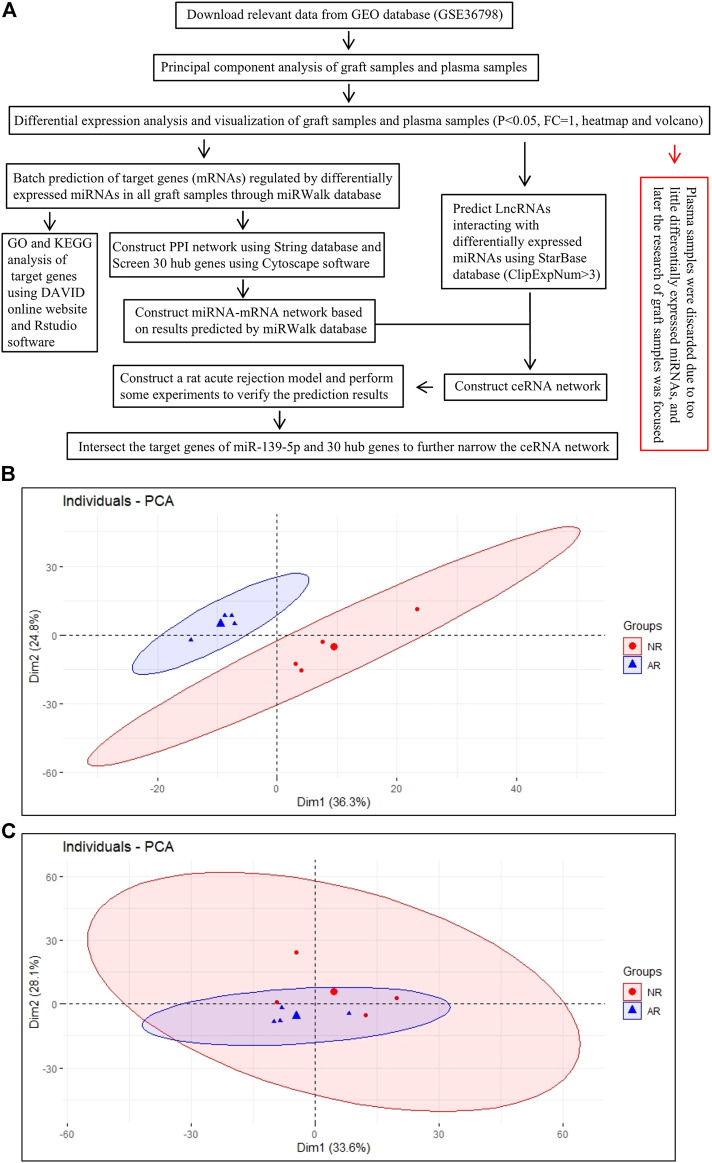
Analysis process of the work. **(A)** Overall work flow chart. **(B)** PCA draft of graft samples.**(C)** PCA draft of plasma samples. Each small dot (triangle or circle) represents one sample, and the large dot represents the average of samples.

PCA, as the name suggests, is to find the most important aspect of the data that is involved in replacing the original data. We aimed to use the idea of dimensionality reduction to convert multiple indicators into a few comprehensive indicators. The samples were clustered according to these principal components. The farther the points representing the samples were on the coordinate axis, the greater the difference between the samples was. As shown in Figure1B, we divided graft samples into two AR groups and one NR group for PCA. The results revealed that the overall distance between different groups was relatively long, indicating that there were major differences among samples on a macro level. Meanwhile, we performed the same analysis on plasma samples, and we found that the difference between plasma groups was relatively smaller than that of the graft (Figure 1C).

### Screening of Differential miRNAs and Their Visualization

**FIGURE 2:**
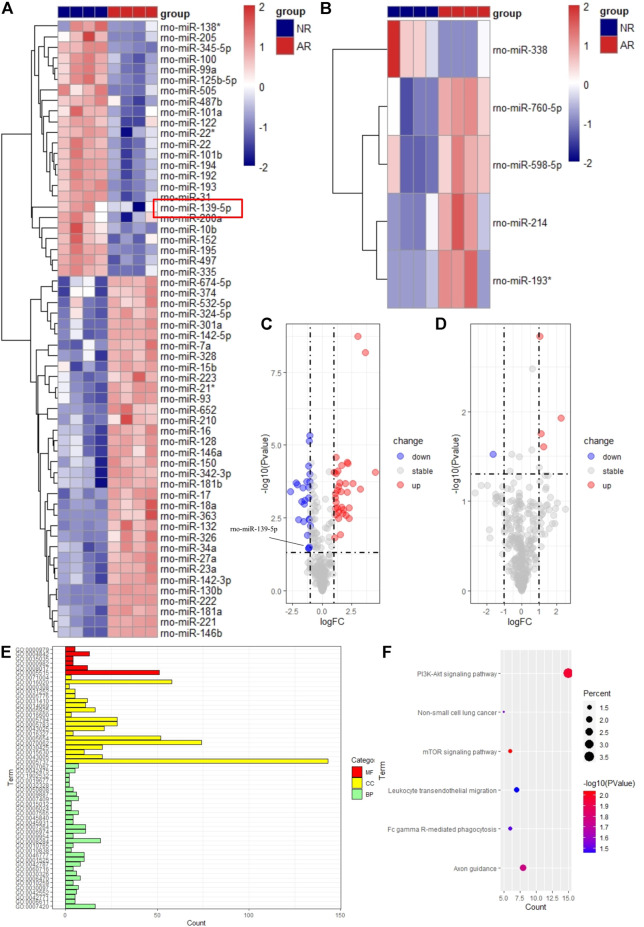
Visualization of differentially expressed miRNAs and functional enrichment analysis of target genes. The heatmap for samples of graft **(A)** and plasma **(B)**. Red means high expression, and blue means low expression. Each column represents a sample, and each row represents an miRNA. The volcano map for samples of graft **(C)** and plasma **(D)**. The abscissa represents logFC, and the ordinate represents the *p*-value. Blue dots represent downregulated miRNAs. Red dots represent upregulated miRNAs. Gray dots represent stable miRNAs. GO **(E)** and KEGG **(F)** analysis of target genes.

After preliminary processing of the GSE36798 dataset, 350 miRNAs accompanied by the grouping information and gene expression matrix were obtained. Finally, we obtained 24 downregulated miRNAs and 34 upregulated miRNAs in the experimental group of transplant samples compared with the control group, according to the screening criteria (Figure 2A). Similarly, there was 1 down-regulated miRNA and 4 up-regulated miRNAs in plasma samples (Figure 2B). All the results we got were visualized with the volcano map and heatmap. In Figures 2A,B, we standardized the expression of all differential miRNAs and visualized them in the form of heatmaps. Whether in graft samples or plasma samples, we found that some miRNAs were downregulated, while other miRNAs were upregulated, indicating obvious differences between different groups. In the volcano map of graft samples, we specially marked rno-miR-139-5p as the candidate miRNA (Figure 2C). Plasma samples were subjected to the same analysis (Figure 2D). Even if the differential expression of miRNAs in plasma samples could possibly help us find biomarkers of ARLT noninvasively, there were too few differentially expressed miRNAs in plasma samples in this work, which led to increasing errors. Therefore, we focused on graft samples and discarded plasma samples in the follow-up work. In summary, the expression profile of miRNAs was significantly different between the AR group and the NR group, which laid the foundation for subsequent analysis.

### Functional Enrichment Analysis for Differentially Expressed miRNAs

To explore which biological behaviors during the occurrence and development of ARLT differentially expressed miRNAs were probably mainly involved in, we used the miRWalk database to predict target genes of differentially expressed miRNAs and performed functional enrichment analysis to indirectly indicate which pathways may be regulated. A total of 58 differentially expressed miRNAs obtained from graft samples were imported into the miRWalk online prediction website, and finally, 11 miRNAs and 416 target genes were exported. Among them, upregulated miRNAs contained miR-142-3p, miR-342-3p, miR-324-5p, miR-532-5p, miR-142-5p, and miR-674-5p and downregulated miRNAs contained miR-345-5p, miR-139-5p, miR-205, miR-335, and miR-125b-5p.

Then, we applied the DAVID online website to perform a functional enrichment analysis for all target genes of differentially expressed miRNAs. The results showed that the main enrichment was focused on BP with 31 terms, and the enrichment of MF and CC, respectively, had 5 and 19 terms in GO analysis (Figure 2E). Among them, one of the GO terms with the most enriched genes was cytoplasm, in which more than 100 genes were enriched, indicating it was very likely that differentially expressed miRNAs were mainly involved in the regulation of ARLT by targeting genes in the cytoplasm. In addition, many immune-related biological processes were enriched in BP, which provided strong evidence for us to further explore the potential mechanism of acute rejection and find more target genes, such as FAM131B, ETNK1, ZFP395, RHEBL1, and PTPN4 (data not shown). In the KEGG analysis, only five pathways were enriched here (Figure 2F). Most genes were enriched in the PI3K-AKT signaling pathway, illustrating that this pathway might play a vital role in the process of acute rejection. At the same time, analysis data revealed that the mTOR signaling pathway and leukocyte transendothelial migration pathway closely related to inflammation and immunity were also enriched and had significant statistical significance.

### Construction of the Related Network

**FIGURE 3:**
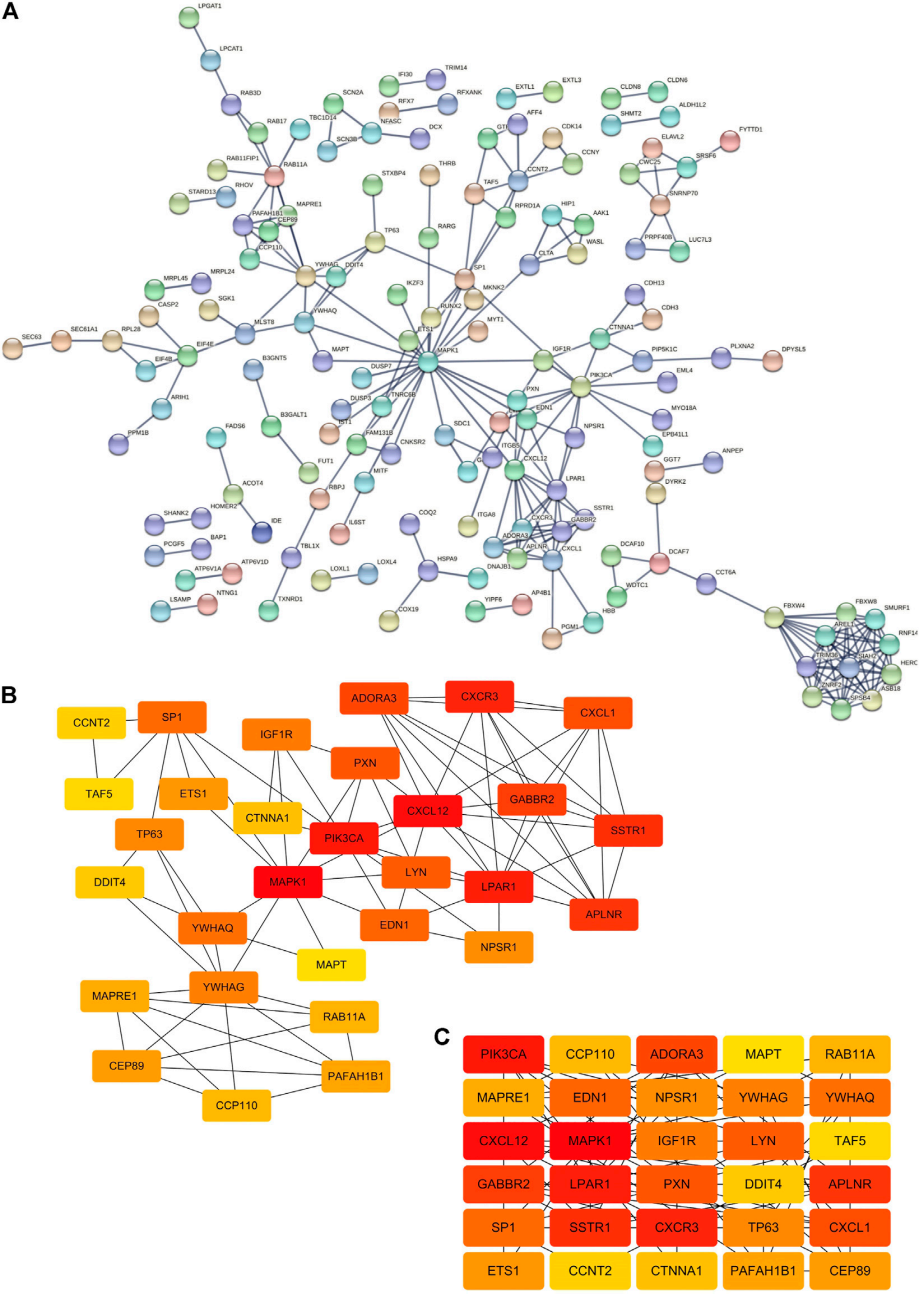
Construction of the PPI network. **(A)** PPI network from the String online website. Each node represents a gene, and each edge represents the interaction between genes. Number of nodes: 395; number of edges: 254; expected number of edges: 211; average node degree: 1.29; PPI enrichment *p*-value: 0.00229. **(B,C)** Top 30 hub genes of the PPI network based on the EPC algorithm in the cytoHubba plugin by Cytoscape software.

**FIGURE 4:**
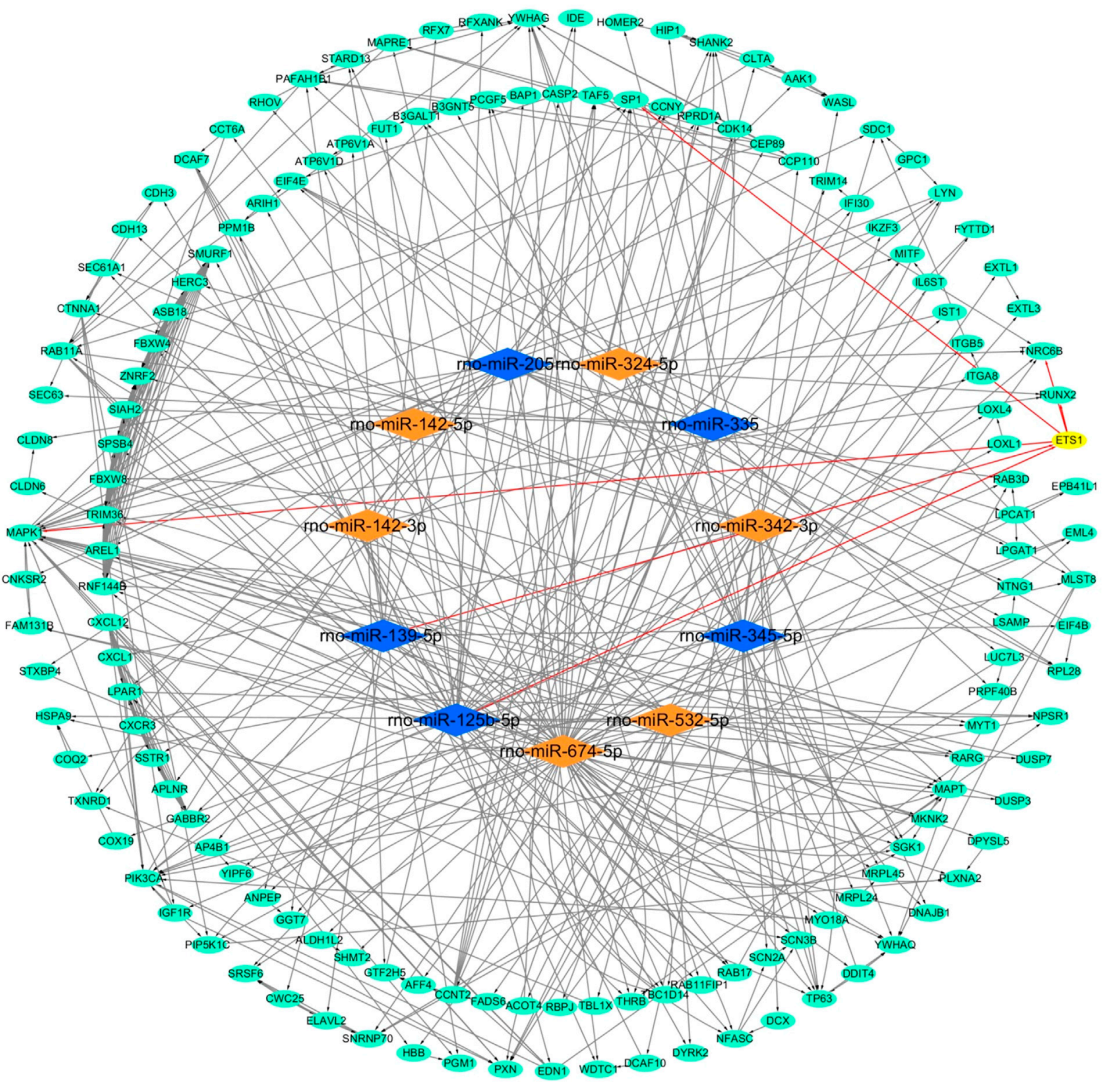
Network of miRNA-mRNA. Orange nodes: upregulated miRNAs. Blue nodes: downregulated miRNAs. Green nodes: target genes. Edges with arrow: miRNAs regulate target genes, or one of the target genes regulates another target gene. One example contains a yellow node and six red edges with an arrow.

For the sake of exploring the interactions between all miRNAs and all target genes, we first constructed a PPI interaction network (Figure 3A). We imported all target genes into the String online website to construct a protein interaction network with the highest confidence level of 0.900 and disconnected nodes hidden. The network contained a total of 395 nodes and 254 edges, and the average node degree was 1.29. Furthermore, we also obtained 30 hub genes via the EPC algorithm in the cytoHubba plug-in by Cytoscape software (Figures 3B,C). Then, we combined miRNAs-target genes and the PPI network to construct a PPI-miRNA interaction network (Figure 4). The network not only shows the regulation of miRNAs on their target genes but also the mutual regulation of different target genes. For instance, the transcription factor ETS1 was not only regulated by rno-miR-125b-5p and rno-miR-139-5p, but it could also regulate MAPK1, SP1, RUNX2, and TNRC6B.

**FIGURE 5:**
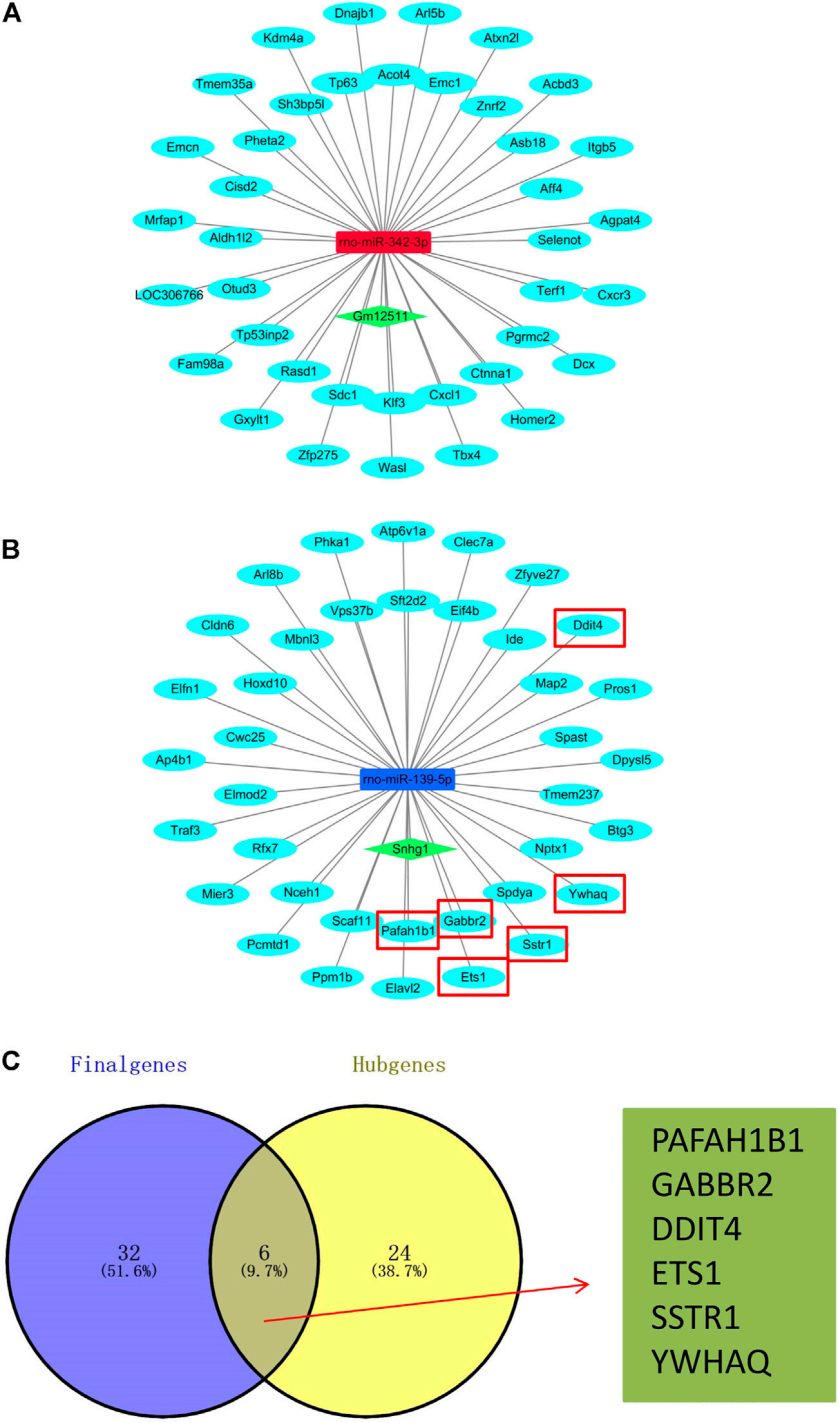
Construction of the ceRNA network. **(A,B)** ceRNA network of the acute rejection model after liver transplantation in rats. Skyblue nodes: mRNAs. Red nodes: upregulated miRNAs. Blue nodes: downregulated miRNAs. Green nodes: lncRNAs. **(C)** Venn diagram of the overlap for target genes (Final genes) regulated by rno-miR-139-5p and 30 hub genes (Hub genes).

After analysis and processing of the aforementioned data, we received the difference information between the AR group and the NR group at the miRNA level and mRNA level. Finally, for the purpose of constructing the entire ceRNA network, we also needed to analyze the difference information at the lncRNA level. We used the lncBase V.2 online prediction website to obtain lncRNAs related to differentially expressed miRNAs and combined them with the miRNA-mRNA network to construct a lncRNA–miRNA–mRNA network (Figures 5A,B). The network mainly consists of two regulatory axes. They were the Gm12511/rno-miR-342-3p/mRNAs axis and the Snhg1/rno-miR-139-5p/mRNAs axis, which indicated that it was very likely that these two regulatory axes functioned in obvious roles in the process of ARLT in rats. We realized that the lncRNA Snhg1/rno-miR-139-5p/mRNAs axis was potentially the ultimate goal of this work *via* subsequent experimental verification. Therefore, we took the intersection of all the mRNAs regulated by rno-miR-139-5p and the 30 hub genes in the aforementioned PPI network to further clarify mRNAs that may participate in the regulation of ARLT, and we finally obtained six key genes, which were, respectively, *PAFAH1B1*, *GABBR2*, *DDIT4*, *ETS1*, *SSTR1*, and *YWHAQ* (Figure 5C).

### Experimental Verification for the ceRNA Network

**FIGURE 6:**
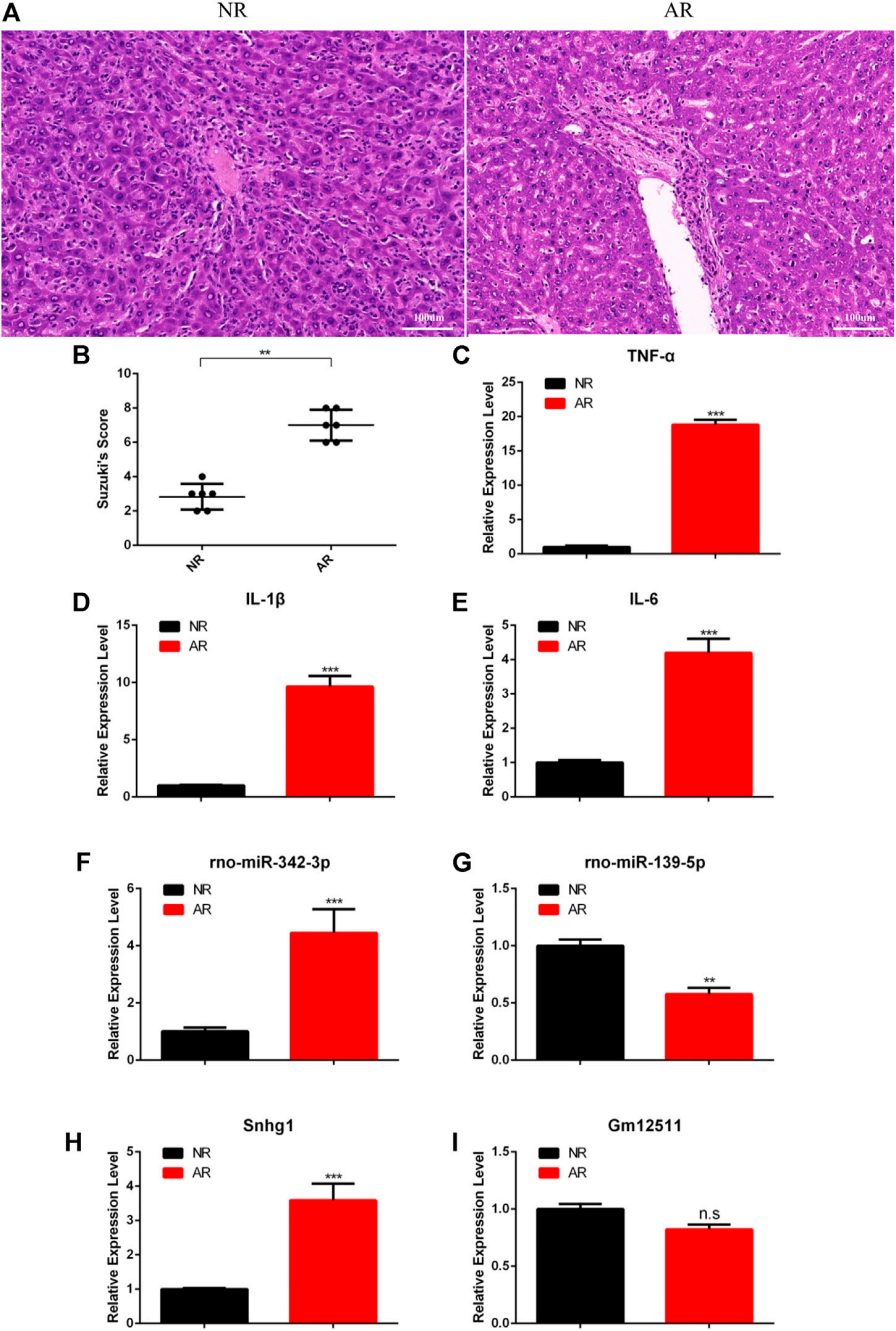
Experimental verification for the ceRNA network. **(A)** HE staining for graft samples after liver transplantation in rats. **(B)** Suzuki’s histological score for tissue damage. **(C–E)** Real-time fluorescence quantitative PCR detection for related inflammatory factors. **(F–I)** Real-time fluorescence quantitative PCR detection for the ceRNA network. All the experiments were performed at least three times, and the data were presented as means ± SD. (^*^ *p* <0.05; ^**^ *p* <0.01; ^***^ *p* <0.001; ns, not significant).

Although we have established the ceRNA network regarding the regulation of ARLT and screened out the most likely regulatory axis, which was still at the stage of conjecture and hypothesis, in order to further verify our conjecture, we established an orthotopic liver transplantation model of acute rejection with xenogeneic rats. In the AR group, Lewis rats were used as donors, and BN rats were used as recipients (Lewis to BN). In the NR group, both donors and recipients were BN rats (BN to BN). The data showed that the liver was more severely damaged in the manifold area in the AR group than the NR group (Figures 6A,B). In addition, we also extracted tissue RNA to detect the expression of related inflammatory factors, such as TNF-α, IL-6, and IL-1β. The results demonstrated that the degree of inflammation was more severe in the AR group (Figures 6C–E). These results indirectly indicated that the aforementioned animal model could be used to verify the ceRNA network we had constructed. So we separately detected the expressions of two regulatory axes screened out previously. The results showed increased rno-miR-342-3p and Snhg1, decreased rno-miR-139-5p, and stable Gm12511 in the AR group (Figures 6F–I). These results illustrate that Snhg1 probably plays a crucial role *via* sequestering rno-miR-139-5p to function as a ceRNA in ARLT.

All in all, we had constructed a ceRNA network that might regulate ARLT and carried out relevant experiments to verify and screen target genes, which deepened our understanding of its potential molecular mechanisms and provided new ideas and possible therapeutic targets for the treatment of related complications after liver transplantation in the future.

## Discussion

At present, the most effective treatment for end-stage liver disease is liver transplantation, and the life quality of patients after liver transplantation is seriously affected by related complications. With the advancement of surgical procedures and the emergence of new drugs, the incidence of complications has been decreased, but it is still very obscure to us about its potential molecular mechanism. Exploring its underlying molecular mechanism will provide more powerful weapons and protection for the treatment of related complications after liver transplantation.

The risk of organ rejection after transplantation is that the body’s immune system can recognize the donor’s antigen and respond. This response is achieved through lymphocyte activation (cell reaction) and antibodies (humoral response). Therefore, looking for genes that are closely connected with immune regulation through high-throughput screening methods may further strengthen the understanding of the mechanism of immune rejection after organ transplantation, thereby improving the efficiency of treatment. With this goal as the starting point, we downloaded data from the GEO database to perform this research and constructed a total of 3 networks, namely, the miRNA–mRNA network, the PPI network, and the ceRNA network. Although we have screened out some possible genes or two regulatory axes from the perspective of bioinformatics, we still need to do further assays to verify our hypothesis. For example, from the ceRNA network we constructed, we learned that the lncRNA Snhg1/rno-miR-139-5p/mRNAs axis probably played an important role in the occurrence and development of ARLT. However, whether this axis is really involved in the regulation of rejection reactions needs more experiments to confirm. Among the aforementioned axes, we held opinions that the axis most likely to be involved in regulation was the Snhg1-miR-139-5p-Mier3 axis. Studies have reported that lncRNA Snhg1 can regulate the differentiation of Treg cells and can affect the immune escape of breast cancer cells by regulating the miR-448/IDO axis (Pei et al., 2018). Choi et al. (2016)found that miR-139-5p, as an effective anti-leukemia molecule, could negatively regulate the proliferation of hematopoietic stem cells and was closely relevant to the occurrence and development of chronic myeloid leukemia. Mier3 has been found to be involved in the regulation of tumor progression (Peng et al., 2017), and it is well known that tumor and immunity are inseparable. Therefore, it can be seen that the Snhg1-miR-139-5p-Mier3 axis is closely associated with immune regulation, which further supports our hypothesis. In addition, we have not verified which of the six target genes we finally screened out were involved in the regulation due to the constraints of time and funding, and we will discuss it in our follow-up work. In addition, Fanbing Meng et al. recently found that the PI3K-AKT signal pathway was engaged in the protection of kidney injury after liver transplantation (Meng et al., 2021). In our work, we also found that this pathway was significantly enriched; then, whether it also plays a prominent role in rejection after liver transplantation deserves further exploration.

In this research, we analyzed the difference between the AR group and the NR group after liver transplantation, trying to excavate potential biomarkers and provide guidance for the clinical treatment of disease. However, we know that there are many factors affecting liver survival after transplantation, and there are many complications, such as thrombosis (Bhangui et al., 2019; Flynn et al., 2019; Bhangui et al., 2020), bile duct anastomotic stricture (Koksal et al., 2017; Sato et al., 2019), viral infection (Di Maira and Berenguer, 2020), and ischemia-reperfusion injury (Zhang et al., 2017; Nakamura et al., 2019). We can also use the same method as in this study to find potential molecular mechanisms of other complications, further enrich strategies and ideas for the treatment of related complications after liver transplantation, and improve the quality of life and the survival rate of patients.

An interesting phenomenon in this work was that we found there were very few differentially expressed miRNAs in plasma samples after we completed the analysis on graft samples and plasma samples, so we discarded these miRNAs in subsequent studies. However, as for why there were so few differentially expressed miRNAs in plasma samples, we thought the main reason was that the source of miRNAs in plasma samples after liver transplantation was from immune cells in the blood rather than liver tissue. The liver is an immune-privileged organ whose immune microenvironment is regulated by both parenchymal and non-parenchymal cells (Thomson et al., 2020), and almost all rodents experience a very low degree of rejection after allogeneic liver transplantation. It is known to us that the main effector cells for rejection are T cells. After circulating T cells enter into the liver, they need to interact with a lot of different cells (antigen-presenting cells, hepatocytes, endothelial cells, etc.) to generate effector T cells (Ronca et al., 2020). During this process, effector T cells may also undergo apoptosis through a caspase3-dependent pathway (Thomson et al., 2020), so very few miRNAs can be secreted into peripheral blood, which may be the reason why there are no ideal positive results in the differential analysis of plasma samples.

In summary, in the network we constructed, we have screened out some targets that may be involved in the regulation of ARLT. Whether it is lncRNA, miRNA, or mRNA, it may play a key role in this process, and it may even become a biomarker for disease treatment and prevention. Even though we have screened out some relevant genes, they are still in the prediction stage at present. We need to further test our hypothesis to make effective contributions to the treatment of clinical diseases in the future.

## Data Availability

The datasets presented in this study can be found in online repositories. The names of the repository/repositories and accession number(s) can be found in the article/Supplementary Material.
