# Supplementary material for: LncRNA Snhg1 Plays an Important Role via Sequestering rno-miR-139-5p to Function as a ceRNA in Acute Rejection After Rat Liver Transplantation Based on the Bioinformatics Analysis
Source: Front Genet. 2022 Jun 2;13:827193. doi: 10.3389/fgene.2022.827193 (PMC9203122; doi:10.3389/fgene.2022.827193)
Supplement: Supplementary file 1 [file DataSheet1.docx]

| GO:0007420 | brain development |
| --- | --- |
| GO:0006611 | protein export from nucleus |
| GO:0042771 | intrinsic apoptotic signaling pathway in response to DNA damage by p53 class mediator |
| GO:0042552 | myelination |
| GO:0030097 | hemopoiesis |
| GO:0010259 | multicellular organism aging |
| GO:0006470 | protein dephosphorylation |
| GO:0030326 | embryonic limb morphogenesis |
| GO:0060716 | labyrinthine layer blood vessel development |
| GO:0042787 | protein ubiquitination involved in ubiquitin-dependent protein catabolic process |
| GO:0001525 | angiogenesis |
| GO:0046777 | protein autophosphorylation |
| GO:0010838 | positive regulation of keratinocyte proliferation |
| GO:0010765 | positive regulation of sodium ion transport |
| GO:0008284 | positive regulation of cell proliferation |
| GO:0009954 | proximal/distal pattern formation |
| GO:0006974 | cellular response to DNA damage stimulus |
| GO:0007264 | small GTPase mediated signal transduction |
| GO:0045931 | positive regulation of mitotic cell cycle |
| GO:0045840 | positive regulation of mitotic nuclear division |
| GO:0007565 | female pregnancy |
| GO:0006024 | glycosaminoglycan biosynthetic process |
| GO:0015012 | heparan sulfate proteoglycan biosynthetic process |
| GO:0007409 | axonogenesis |
| GO:0009887 | organ morphogenesis |
| GO:0050808 | synapse organization |
| GO:0032328 | alanine transport |
| GO:0019677 | NAD catabolic process |
| GO:1902532 | negative regulation of intracellular signal transduction |
| GO:0042475 | odontogenesis of dentin-containing tooth |
| GO:0007067 | mitotic nuclear division |
| GO:0005737 | cytoplasm |
| GO:0043005 | neuron projection |
| GO:0015630 | microtubule cytoskeleton |
| GO:0030425 | dendrite |
| GO:0070062 | extracellular exosome |
| GO:0005654 | nucleoplasm |
| GO:0016327 | apicolateral plasma membrane |
| GO:0043025 | neuronal cell body |
| GO:0005783 | endoplasmic reticulum |
| GO:0005794 | Golgi apparatus |
| GO:0016600 | flotillin complex |
| GO:0005925 | focal adhesion |
| GO:0014069 | postsynaptic density |
| GO:0031410 | cytoplasmic vesicle |
| GO:0005776 | autophagosome |
| GO:0031252 | cell leading edge |
| GO:0000308 | cytoplasmic cyclin-dependent protein kinase holoenzyme complex |
| GO:0016020 | membrane |
| GO:0071004 | U2-type prespliceosome |
| GO:0005515 | protein binding |
| GO:0008017 | microtubule binding |
| GO:0000982 | transcription factor activity, RNA polymerase II core promoter proximal region sequence-specific binding |
| GO:0035035 | histone acetyltransferase binding |
| GO:0004842 | ubiquitin-protein transferase activity |
| GO:0000979 | RNA polymerase II core promoter sequence-specific DNA binding |
